# Ontology-based *Brucella *vaccine literature indexing and systematic analysis of gene-vaccine association network

**DOI:** 10.1186/1471-2172-12-49

**Published:** 2011-08-26

**Authors:** Junguk Hur, Zuoshuang Xiang, Eva L Feldman, Yongqun He

**Affiliations:** 1 Bioinformatics Program, University of Michigan, Ann Arbor, MI 48109, USA; 2 Department of Neurology, University of Michigan, Ann Arbor, MI 48109, USA; 3 Unit for Laboratory Animal Medicine, University of Michigan, Ann Arbor, MI 48109, USA; 4 National Center for Integrative Biomedical Informatics, University of Michigan, Ann Arbor, MI 48109, USA; 5 Department of Microbiology and Immunology, University of Michigan, Ann Arbor, MI 48109, USA

## Abstract

**Background:**

Vaccine literature indexing is poorly performed in PubMed due to limited hierarchy of Medical Subject Headings (MeSH) annotation in the vaccine field. Vaccine Ontology (VO) is a community-based biomedical ontology that represents various vaccines and their relations. SciMiner is an in-house literature mining system that supports literature indexing and gene name tagging. We hypothesize that application of VO in SciMiner will aid vaccine literature indexing and mining of vaccine-gene interaction networks. As a test case, we have examined vaccines for *Brucella*, the causative agent of brucellosis in humans and animals.

**Results:**

The VO-based SciMiner (VO-SciMiner) was developed to incorporate a total of 67 *Brucella *vaccine terms. A set of rules for term expansion of VO terms were learned from training data, consisting of 90 biomedical articles related to *Brucella *vaccine terms. VO-SciMiner demonstrated high recall (91%) and precision (99%) from testing a separate set of 100 manually selected biomedical articles. VO-SciMiner indexing exhibited superior performance in retrieving *Brucella *vaccine-related papers over that obtained with MeSH-based PubMed literature search. For example, a VO-SciMiner search of "live attenuated *Brucella *vaccine" returned 922 hits as of April 20, 2011, while a PubMed search of the same query resulted in only 74 hits. Using the abstracts of 14,947 *Brucella*-related papers, VO-SciMiner identified 140 *Brucella *genes associated with *Brucella *vaccines. These genes included known protective antigens, virulence factors, and genes closely related to *Brucella *vaccines. These VO-interacting *Brucella *genes were significantly over-represented in biological functional categories, including metabolite transport and metabolism, replication and repair, cell wall biogenesis, intracellular trafficking and secretion, posttranslational modification, and chaperones. Furthermore, a comprehensive interaction network of *Brucella *vaccines and genes were identified. The asserted and inferred VO hierarchies provide semantic support for inferring novel knowledge of association of vaccines and genes from the retrieved data. New hypotheses were generated based on this analysis approach.

**Conclusion:**

VO-SciMiner can be used to improve the efficiency for PubMed searching in the vaccine domain.

## Background

Since the introduction of Edward Jenner's vaccine against smallpox in 1796, vaccines have proven useful in their ability to stimulate the immune system and confer protection against infections by pathogenic microorganisms. As such, vaccines provide safe, effective, and cost-effective means to reduce the incidence of infectious diseases. Vaccine research and development has undergone a renaissance in recent years. This is in part attributable to the cost-effectiveness of vaccines and advanced post-genomic technologies [1]. Infectious diseases remain a major source of morbidity and mortality worldwide, causing 14.7 million deaths (26% of total global mortality) in 2001 [2]. Although considerable progress has been made, vaccination against many medically important pathogens, such as Human immunodeficiency virus (HIV) and *Brucella*, has been unsuccessful due to their unique mechanisms of pathogenesis [3,4]. For rational vaccine design, extensive research is required to better understand the pathogenesis and protective immune responses against such diseases [5].

As the field of vaccine research continues to grow, the amount of vaccine literature is rapidly increasing. Search of the biomedical literature database PubMed (http://www.ncbi.nlm.nih.gov/pubmed) revealed the number of vaccine-related citations has almost doubled over the last decade (from 99,316 papers before 2000 to over 196,055 papers as of April 20, 2011). Due to this rapid growth of published information, it is no longer feasible to keep up to date with all the new literature manually, even within one's own research area. The field of literature mining, a means of computer-assisted information extraction from literature data, is becoming increasingly important to cope with the expanding volume of available biomedical literature. Articles in PubMed are indexed with NLM-developed Medical Subject Headings (MeSH), which is a controlled vocabulary of over 25,000 terms organized in a hierarchical fashion with 15 top-level categories [6]. New articles deposited into PubMed will be read by human experts and indexed with relevant MeSH terms to represent the content. The use of MeSH indexing in PubMed provides a consistent way to retrieve information that may use different terminologies for the same concepts. However, MeSH does not thoroughly cover many biomedical domains, including the vaccine domain. For example, "*Brucella *vaccine" is currently the lowest level term under 'vaccines' in the MeSH hierarchy. No individual *Brucella *vaccines are currently indexed by MeSH. Furthermore, as a controlled vocabulary, instead of a biomedical ontology, MeSH does not contain logic definitions for the relations between different terms.

A biomedical ontology represents the consensus-based controlled vocabularies of terms and relations, which are logically formulated to promote intelligent information retrieval and modeling. The Vaccine Ontology (VO; http://www.violinet.org/vaccineontology) [7] is a community-based ontology in the domain of vaccine and vaccination. VO classifies existing vaccines in licensed use, on trial, or in research. The relations between different VO terms have been logically defined and support advanced semantic reasoning. SciMiner is a web-based literature mining tool developed for target (gene and protein) identification and as well for functional enrichment analysis [8,9]. SciMiner uses dictionary and rule-based approaches for literature mining. We hypothesized that a VO-based SciMiner literature mining program would dramatically improve vaccine literature indexing and support literature-based discovery of vaccine-gene interaction networks.

As a case study, we have focused on *Brucella*, an intracellular bacterium that causes brucellosis, the most common zoonotic disease worldwide [10]. Using the VIOLIN vaccine database and analysis system [11], we previously studied *Brucella *vaccines using multiple bioinformatics approaches [12]. This study introduced the manual curation of *Brucella *vaccines in the VIOLIN database and in the VO and the online query system of annotated *Brucella *vaccine information. However, there has not been a systematic study on how to use the curated *Brucella *vaccine terms and hierarchy in VO for natural language processing (NLP)-based indexing of *Brucella *vaccine literature, analysis of vaccine-associated *Brucella *genes, and the prediction of the interaction network between vaccines and *Brucella *genes. Such study would significantly improve our understanding of *Brucella *vaccinology and provide a use case for ontology-based text mining in the field of vaccinology and immunology.

In this study, a VO-based SciMiner approach (VO-SciMiner) was developed, 1) to retrieve and index vaccine names and *Brucella *genes from PubMed articles, and 2) further identify and analyze interactions between *Brucella *vaccines and *Brucella *genes. These studies revealed that VO-SciMiner efficiently indexed *Brucella *vaccine publications and generated a comprehensive vaccine-gene interaction network.

## Methods

### *Brucella *vaccine terms and their semantic relations

All *Brucella *vaccine terms and their semantic relations available in VO (http://www.violinet.org/vaccineontology) were used in this study. The current version consisting of 67 *Brucella *VO terms (as of April 2011) and their synonyms were added to the dictionary of VO-SciMiner.

### Term expansion rules of *Brucella *VO terms in SciMiner

A training set consisting of 90 papers with manually confirmed *Brucella *vaccine VO terms was used to optimize SciMiner for VO term identification. Patterns of variations of VO terms in the actual text were learned and implemented in VO-SciMiner as a series of term expansion rules to maximize the power of term identification. Examples of rules are below:

(1) Variations of functional words are used. For example, "encoding" in the vaccine name can be replaced with "coding for".

(2) Unabbreviated gene names can be used instead of abbreviated gene symbols (*e.g*., trigger factor for TF).

(3) VO terms with a full pathogen name (*e.g*., *Brucella abortus *strain 19) can be abbreviated (*e.g*., *B. abortus *strain 19) or abbreviated names can be fully spelled out.

(4) The word "strain(s)" can be added. For example, "*Brucella melitensis *strain Rev. 1" can be an alternative form for "*Brucella melitensis *Rev. 1".

(5) Pathogen names can be removed. For example, "Rev. 1" is frequently used without "*Brucella melitensis*" in the text for "*Brucella melitensis *Rev. 1".

(6) Spaces and special characters can be altered. For example, "Rev.1", "Rev1", "Rev-1" and "Rev-I" can be used as additional terms for "Rev. 1".

(7) Any SciMiner-identified VO term is ignored if a corresponding genus or disease name does not appear in the text. SciMiner reports identified *Brucella *VO terms only if there is at least one occurrence of "*Brucella*" or "brucellosis" in any part of the text. This restriction helps to improve the precision of the analysis by removing potentially non-specific identification from other species.

### Performance evaluation of VO-SciMiner

To evaluate the performance of VO-SciMiner in literature indexing, 50 negative and 50 positive papers were manually compiled. Each paper in the positive set had at least one confirmed *Brucella *VO term and contained a total of 89 confirmed paper-VO associations, defined as the "gold standard". The paper set lacking *Brucella *vaccine terms, but still in the domain of *Brucella *or brucellosis, was used as a negative control. Performance was measured by recall, precision, and F-measure (defined below), where the paper-VO associations that matched the gold standard constituted true positives (TP), associations that did not match were false positives (FP), and the gold standard associations that were not matched were false negatives (FN) [13].

$$Recall\;\left(R\right)=TP∕\left(TP+FN\right)$$

$$Precision\;\left(P\right)=TP∕\left(TP+FP\right)$$

$$F-measure=\left(2*P*R\right)∕\left(P+R\right)$$

### Indexing of PubMed abstracts with *Brucella *VO terms using VO-SciMiner

In order to assess the improved information retrieval using ontology-based indexing, VO-SciMiner was applied to a subset of PubMed abstracts. For the present analyses, a total of 14,947 *Brucella *related papers (defined by "*Brucella *OR brucellosis" in PubMed as of April 20, 2011) were processed and indexed by SciMiner. The results were compared with PubMed search results using the corresponding VO terms as queries.

### *Brucella *gene name identification from literature

The NCBI RefSeq database contains 10 fully sequenced and annotated *Brucella *genomes, which share more than 95% sequence homology [14]. However, since these genomes have been independently annotated by multiple research groups, orthologous genes from various *Brucella *genomes frequently have different gene names (including synonyms) and annotations. To retrieve all relevant literature associated with individual *Brucella *genes, we used OrthoMCL-DB [15] to identify the orthologs among different *Brucella *genomes. After removing redundant terms, gene names and annotations were merged for individual orthologs. For genes with variable names across different *Brucella *genomes, the most frequently used gene name was selected as the representative name; other names were included as synonyms.

### Identification of protein localization and over-represented biological functions

The protein subcellular localization of these genes was predicted by our reverse vaccinology program Vaxign [16], which employs PSORTb 3.0, an open-source software for subcellular localization prediction with high accuracy (precision values > 97% and recall values > 94% based on benchmark evaluations) [17]. To determine the over-represented roles of identified *Brucella *genes associated with *Brucella *vaccines, an enrichment analysis using the Cluster of Orthologous Group (COG, http://www.ncbi.nlm.nih.gov/COG) information was employed [10,18]. Fisher's exact [19] test was used to calculate the statistical significance of these COG functional groups (p-value < 0.05).

### Identification of *Brucella *vaccines and genes interaction Network

VO-SciMiner was applied to 14,947 *Brucella *papers to identify *Brucella *genes and vaccines (VO). If a *Brucella *gene name and a VO vaccine term were identified in the same document (at the abstract level), a co-citation connection was established between the gene and vaccine. The complete *Brucella *gene-vaccine network was visualized in Cytoscape [20], an open source bioinformatics software for visualizing molecular interaction networks. Each gene name and VO vaccine term is represented as a node in the network. The number of papers for each co-citation is represented as thickness of each edge.

### Web-based implementation of VO-SciMiner

A VO-SciMiner website (http://www.violinet.org/vo-sciminer) has been developed to allow users to search *Brucella *vaccine papers indexed with VO terms. This website uses PHP on a Linux web server. A MySQL database server is used to store network information. A virtuoso Resource Description Framework (RDF) store is used to store the contents of VO, including asserted and inferred VO hierarchies. A *cron *(a time-based job scheduler) job has been set up on the web server to retrieve new *Brucella*-related publications from PubMed on a weekly basis. Once retrieved, the publications will be processed through the VO-SciMiner analysis workflow, and the results will be placed in the MySQL database.

## Results

### Overall workflow of VO-based literature mining approach

**Figure 1:**
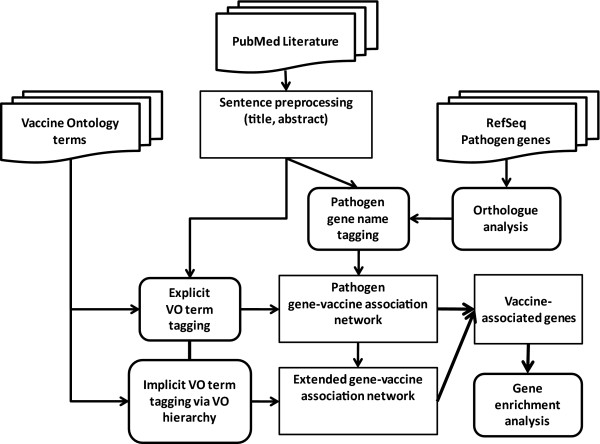
**Overall VO-SciMiner workflow**.

Figure 1 illustrates the proposed workflow of the VO-SciMiner system. Pathogen-related literature is collected through PubMed. The titles and abstracts of the retrieved documents are pre-processed by a sentence splitter, and analyzed by the VO-SciMiner to identify vaccine VO terms and pathogen gene names. The associations of these vaccines and pathogen genes are further analyzed via a vaccine-gene network visualized with Cytoscape. Over-represented biological functions of pathogen genes are examined using the COG information.

### Asserted and inferred VO hierarchies

The design of VO is based on OBO (Open Biological and Biomedical Ontologies) Foundry Principles [21]. VO utilizes the Basic Formal Ontology (BFO), a domain-independent ontology, as an upper level ontology [7]. The relation terms defined in the Relation Ontology (RO) are used in VO for representing commonly used relationships. Each subclass found in VO has an "*is_a*" relationship with its parent class. This characteristic ensures that all vaccine subclasses (*e.g*., *Brucella *RB51) are included when a parent class (*e.g*., "*Brucella *vaccine") is searched.

**Figure 2:**
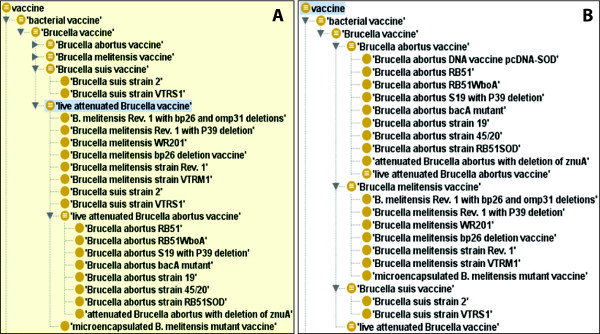
***Brucella *vaccines in VO**. (A) Asserted hierarchy; (B) Inferred hierarchy. These are Protégé screenshots of VO without (A) or with reasoning using HermiT 1.3.2 (B).

Different types of vaccines are classified based on the asserted VO vaccine hierarchy (Figure 2A) and inferred VO vaccine hierarchy (Figure 2B). The asserted ontology hierarchy is an ontology hierarchy specified by ontology developers. The inferred ontology hierarchy, based on the Web Ontology Language (OWL) [20], is generated by a specific ontology reasoner, such as HermiT (http://hermit-reasoner.com). An ontology reasoner infers logical consequences from a set of asserted facts based on necessary and sufficient conditions. If an ontology term is a member of an ontology class, then the term must fulfill the class's *necessary *conditions. Some classes may also have sufficient conditions, and in those cases the combination of necessary and sufficient conditions makes an ontology class a fully defined class. For example, the class term *live attenuated vaccine *is fully defined as [*'live vaccine' and (has_quality some attenuated)*]. Many vaccines have the characteristics (necessary condition) of being attenuated. For example, RB51 has the following necessary condition [*has_quality some attenuated*]. RB51 and many other vaccines are also live vaccines (another necessary condition). These two necessary conditions are sufficient to define *live attenuated vaccine*. Therefore, based on the logical definition, the VO term RB51 can be inferred as a *live attenuated vaccine*. Since RB51 is also a *Brucella *vaccine, it can be further inferred as a *live attenuated Brucella vaccine*. Such classification can only be obtained by an ontology inferring since RB51 is not originally asserted as such (Figure 2A).

### Performance evaluation of VO term identification using VO-SciMiner

To test the performance of SciMiner, 50 negative and 50 positive papers were manually compiled. The positive set had 89 confirmed paper-VO associations, whereas the negative set contained no VO terms. In this testing set of 100 papers, VO-SciMiner identified 82 paper-VO associations, of which 81 belonged to the gold standard set, thereby achieving 91% recall, 99% precision, and 95% F-measure (Table 1).

**Table 1:** Performance of VO-SciMiner literature mining

Testing set	Gold-Standard VO-Paper association	Identified by VO-SciMiner	True Identification	Recall	Precision	F-measure
Positive 50	89	81	81	91%	100%	95%
Negative 50	0	1	0			

Total	89	82	81	91%	99%	95%

### VO-based indexing of *Brucella *vaccine papers using VO-SciMiner

Literature indexing provides researchers with a means to navigate efficiently through a network of scholarly scientific articles in a subject domain. VO-SciMiner was employed to index *Brucella *papers with *Brucella *vaccine ontologies. For comparison purposes, typical PubMed searches were conducted using corresponding terms (Table 2). When no hierarchical VO structure information was used, SciMiner performed in a manner similar to that of a typical PubMed search. For example, the query of "*Brucella *vaccine" yielded 1,379 hits in PubMed Entrez and 1,359 hits in SciMiner. However, when the hierarchical structure of VO was used, VO-SciMiner returned significantly more papers than the typical PubMed searches. A PubMed search of "live attenuated *Brucella *vaccine" returned 74 hits, while the VO-based SciMiner search returned 922 hits. The primary reason for this increased number of hits is due to the addition of papers indexed with the child (subclass) vaccine terms, belonging to "live attenuated *Brucella *vaccines" in the VO hierarchy (Figure 2). For some vaccine terms, the VO-based SciMiner search increased the number of positive PubMed paper hits by up to 15-fold (Table 2).

**Table 2:** VO-based indexing results using VO-SciMiner and PubMed (as of April 20, 2011)

		VO-SciMiner without child VOs		VO-SciMiner with child VOs	
		
PubMed Search Keywords	PubMed Entrez	Total (only by VO-SciMiner)	Common	Total (only by VO-SciMiner)	Common
*Brucella *vaccine	1,379	1,359 (0)	1,359	2,155 (790)	1,365
Live attenuated *Brucella *vaccine	74	85 (12)	73	922 (849)	73
Live attenuated *Brucella abortus *vaccine	52	50 (1)	49	736 (647)	49
Live attenuated *Brucella *melitensis vaccine	36	37 (1)	36	204 (168)	36
Live attenuated *Brucella suis *vaccine	5	5 (0)	5	23 (18)	5

**Figure 3:**
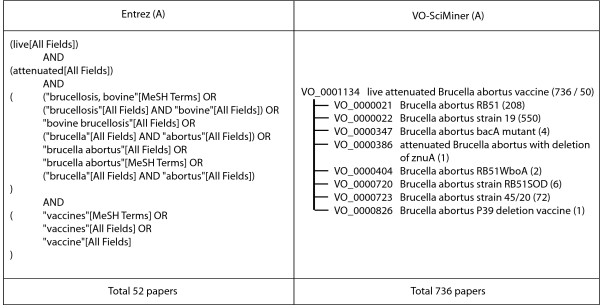
**Comparison between the Entrez system and VO-based literature search approaches**. An example of searching "live attenuated *Brucella abortus *vaccine" is illustrated.

The differences in literature retrieval can be explained by the differences in how query terms are interpreted. The PubMed Entrez system interprets the query "live attenuated *Brucella *vaccines" using MeSH-based querying system (Figure 3). VO-SciMiner does not perform such a complicated search. It uses the hierarchical structure of VO to include any subclass terms of a given query. MeSH in the PubMed also has such a feature, but does not include detailed vaccine terminologies in its vocabulary. In addition, as a formal ontology, VO also has logical semantic relations that allow formation of inferred classes for more advanced reasoning.

PubMed searches generally yielded high precision for most of the specific *Brucella *vaccines (data not shown). In some cases, however, our VO-based approach was able to significantly improve the precision. For example, a typical PubMed search using "*Brucella suis *(strain 2 OR S2)" had 63 hits as of April 20, 2011, while a corresponding VO (VO_0000722; *B. suis *strain 2) was identified in only 18 out of 14,947 *Brucella *related papers. Manual review of the results revealed that the PubMed search had only 12 true positives out of 63 hits, while VO-SciMiner indexing had 17 true positives out of 18 hits. This result demonstrates a significantly improved precision can be obtained using VO-based SciMiner indexing.

### Identification of *Brucella *vaccine-associated genes

From 14,947 *Brucella*-related papers, 1,009 distinct interactions between 51 *Brucella *vaccine VO terms and 140 *Brucella *genes were identified. Grouped based on their functional annotations, the details of these 140 *Brucella *genes and their various annotations are listed in Table 3. Based on sub-cellular localization annotation, these 140 *Brucella *proteins are located in different sub-cellular areas, including outer membrane (10 proteins), cytoplasm (75 proteins), periplasm (10 proteins), cytoplasmic membrane (25 proteins), extracellular matrix (2 proteins) and unknown location (18 proteins).

**Table 3:** *Brucella *genes in the gene-VO network

Locus Tag	Gene symbol	Gene name	# of associated VO terms	# of papers - All VO terms (Live Attenuated vaccines)	Virulence factor or Protective antigen	Sub-cellular localization (PSORTb 3.0 score)
**Nucleotide transport and metabolism**						

BMEI0296	purE	phosphoribosylaminoimidazole carboxylase, catalytic subunit	11	4 (4)	V	U (2.00)
BMEI0295	purK	phosphoribosylaminoimidazole carboxylase ATPase subunit	4	1 (1)		CM (7.88)

**Signal transduction mechanisms**						

BMEI2035	bvrS	sensor histidine kinase BvrS, putative	10	3 (1)	V	CM (10.00)
BMEI0190	ptsP	phosphoenolpyruvate-protein phosphotransferase	5	1 (1)		C (9.97)
BMEI2036	bvrR	DNA-binding response regulator BvrR, putative	10	4 (2)	V	C (9.97)

**Cell motility**						

BMEII0150	fliC	flagellin family protein	5	3 (0)	V	E (9.71)

**Transcription**						

BMEII0665	rnr	exoribonuclease, VacB/RNase II family	6	1 (1)		C (9.97)
BMEII0427	eryD	erythritol transcriptional regulator	2	1 (0)		C (8.96)
BMEII1116	vjbR	transcriptional regulator, LuxR family	10	3 (2)	V	U (2.00)
BMEI0749	rpoB	DNA-directed RNA polymerase subunit beta	6	5 (1)		C (9.97)
BMEI1649	ureG-1	urease accessory protein UreG	2	1 (0)		C (9.97)
BMEI2036	bvrR	DNA-binding response regulator BvrR, putative	10	4 (2)	V	C (9.97)

**Amino acid transport and metabolism**						

BMEII0404	leuB	3-isopropylmalate dehydrogenase	5	1 (1)		C (9.97)
BMEI0101	cysK	Cysteine synthase A	6	1 (1)	V	C (9.26)
BMEI1653	ureB-1	urease subunit beta	3	2 (0)		C (9.26)
BMEII0561	gcvP	glycine dehydrogenase	5	1 (0)	V	C (9.97)
BMEI1654	ureA-1	urease subunit gamma	3	2 (0)		C (9.26)
BMEII0193	potA	ABC transporter	6	3 (2)		C (9.12)
BMEII1054	hisG	ATP phosphoribosyltransferase catalytic subunit	2	1 (0)		C (9.97)
BMEII0205	dppF	ABC transporter	6	3 (2)		CM (7.88)
BMEI1324	pepN	aminopeptidase N	5	1 (1)	V	C (9.12)
BMEII0407	asd	aspartate-semialdehyde dehydrogenase	6	1 (0)		C (9.97)
BMEI1652	ureC-1	urease subunit alpha	3	3 (0)		C (9.97)
BMEI0933	cysK	cysteine synthase A	6	1 (1)	V	C (9.97)

**Secondary metabolites biosynthesis, transport and catabolism**						

BMEI1111	acpXL	acyl carrier protein	5	2 (0)		C (9.97)
BMEI1475	acpP	acyl carrier protein	5	1 (0)		C (9.26)
BMEI0546	pncA	pyrazinamidase/nicotinamidase	2	1 (0)	V	C (9.97)

**Cell wall/membrane/envelope biogenesis**						

BMEI1829	ropB	outer membrane protein, putative	23	37 (17)		OM (10.00)
BMEI1237	galE	epimerase/dehydratase family protein, putative	6	3 (1)	V	C (8.96)
BMEI1249	omp25	outer-membrane protein Omp25	11	17 (4)	V, P	OM (10.00)
BMEI0402	omp31-1	outer membrane protein Omp31	23	22 (9)	P	OM (10.00)
BMEII0844	omp31-2	outer membrane protein, 31 kDa	19	17 (7)		OM (10.00)
BMEI1413	gmd	GDP-mannose 4,6-dehydratase	5	2 (0)	V	C (9.97)
BMEI0997	wbdA	glycosyl transferase, group 1 family protein	16	3 (1)	V	U (2.00)
BMEI1416	rfbE	O-antigen export system ATP-binding protein RfbE	6	3 (2)		CM (7.88)
BMEII0847	wbjE	putative glycosyltransferase	5	1 (0)		U (2.00)
BMEI1393	wbpZ	glycosyl transferase, group 1 family protein	5	1 (0)	V	C (9.26)
BMEI1335	omp	outer membrane lipoprotein-related protein	12	5 (2)		U (2.00)
BMEI0998	wboA	glycosyl transferase WboA	17	16 (10)	V	C (8.96)
BMEI0340	pal	lipoprotein, Pal family	12	7 (3)	P	OM (10.00)
BMEI0830	yaeT	bacterial surface antigen	23	37 (17)		OM (10.00)
BMEI1417	wbkB	wbkB protein	5	2 (0)	V	U (2.00)
BMEI0509	lpcC	lipopolysaccharide core biosynthesis mannosyltransferase LpcC	9	2 (1)		C (9.97)
BMEI1414	perA	perosamine synthase, putative	10	3 (1)	V	C (9.97)
BMEII0837	hyaD	glycosyl transferase, group 2 family protein	5	1 (0)		CM (9.82)
BMEI1404	wbkA	mannosyltransferase, putative	16	7 (3)	V	C (9.26)
BMEII0253	mepA	penicillin-insensitive murein endopeptidase	5	1 (1)		P (9.76)

**Energy production and conversion**						

BMEII0404	leuB	3-isopropylmalate dehydrogenase	5	1 (1)		C (9.97)
BMEI0474	petB	ubiquinol-cytochrome c reductase, cytochrome b	12	4 (2)		CM (10.00)
BMEII0429	eryB	glycerol-3-phosphate dehydrogenase	2	1 (0)	V	C (9.97)
BMEI0137	mdh	malate dehydrogenase	5	1 (1)		U (4.99)
BMEI0140	kgd	alpha-ketoglutarate decarboxylase	11	8 (4)		C (9.97)
BMEII0076	tycC	enterobactin synthetase, component F, putative	2	1 (0)		C (9.97)
BMEI1547	atpI	ATP sythase protein I, putative	6	1 (1)		U (2.00)

**Replication, recombination and repair**						

BMEI0147	xerC	site-specific tyrosine recombinase XerC	5	1 (0)		C (9.26)
BMEI0215	ialA	dinucleoside polyphosphate hydrolase	1	1 (0)		C (9.97)
BMEI0884	gyrA	DNA gyrase subunit A	2	1 (0)		C (9.97)
BMEI0040	xerD	site-specific tyrosine recombinase XerD	5	2 (0)		C (9.97)
BMEI0880	ssb	single-stranded DNA-binding protein family	5	2 (1)		C (9.26)
BMEI1823	gyrB	DNA gyrase subunit B	2	1 (0)		C (9.97)
BMEI1200	parC	DNA topoisomerase IV subunit A	2	1 (0)		C (9.12)
BMEI0878	uvrA	excinuclease ABC subunit A	5	2 (1)	V	C (9.97)
BMEI1307	xerC	site-specific recombinase, phage integrase family	5	1 (0)		C (9.97)
BMEII0676	parE	DNA topoisomerase IV subunit B	2	1 (0)		C (9.97)
BMEI1946	mutM	formamidopyrimidine-DNA glycosylase	1	1 (0)	V	C (9.97)
BMEII0739	alkB	alkylated DNA repair protein AlkB	6	1 (1)		U (2.00)
BMEII0184	insN	IS3 family element, transposase orfA	6	1 (1)		U (2.00)
BR1202	recA	recombinase A	2	1 (0)	V	C (10.00)

**Posttranslational modification, protein turnover, chaperones**						

BMEI1650	ureF	urease accessory protein UreF, putative	2	1 (0)		U (2.00)
BMEII1047	groES	co-chaperonin GroES	1	1 (0)		C (9.97)
BMEI1041	sufC	ABC transporter, ATP-binding protein	6	3 (2)		C (9.97)
BMEI2001	dnaJ	chaperone protein DnaJ	1	1 (0)		C (9.97)
BMEI1330	htrA	serine protease	7	6 (3)	V	P (9.76)
BMEI2002	dnaK	molecular chaperone DnaK	12	7 (2)	V, P	C (9.97)
BMEII1048	groEL	chaperonin GroEL	10	9 (5)		C (9.97)
BMEI1655	ureD-1	urease accessory protein UreD	3	2 (0)		C (9.26)
BMEI1649	ureG-1	urease accessory protein UreG	2	1 (0)		C (9.97)
BMEII0401	trx-2	thioredoxin	5	2 (1)		C (9.26)
BMEI1069	tig	trigger factor	10	2 (0)	V, P	C (8.96)
BMEI1651	ureE	urease accessory protein UreE	2	1 (0)		C (9.97)
BMEI1060	dsbA	outer membrane protein, putative	23	37 (17)	V	U (2.00)
BMEI2022	trx-1	thioredoxin	5	2 (1)		C (9.26)
BMEI1265	surA	peptidyl-prolyl cis-trans isomerase, putative	9	1 (1)	P	P (9.76)
BMEI1492	exsA	ABC transporter, ATP-binding/permease protein	6	1 (1)	V	CM (10.00)

**Translation, ribosomal structure and biogenesis**						

BMEI2010	infC	translation initiation factor IF-3	2	2 (0)		C (9.97)
BMEI0826	frr	ribosome recycling factor	5	2 (1)		C (9.97)
BMEI0748	rplL	50S ribosomal protein L7/L12	16	17 (6)	P	U (6.49)
BMEI0752	rpsL	30S ribosomal protein S12	5	1 (1)		C (9.26)
BMEI1497	tlyA	hemolysin A	1	2 (0)		C (8.96)

**Inorganic ion transport and metabolism**						

BMEII0003	modC	molybdenum ABC transporter, ATP-binding protein	6	3 (2)		C (9.12)
BMEII0893	katA	catalase	6	2 (0)		P (10.00)
BMEII0177	znuC	zinc ABC transporter, ATP-binding protein	6	3 (2)	V	CM (7.88)
BMEI0790	phoA	bacterial alkaline phosphatase	9	7 (2)		P (10.00)
BMEII0581	sodC	superoxide dismutase, Cu-Zn	15	14 (8)	V, P	P (10.00)
BMEI1292	fsr	fosmidomycin resistance protein	2	1 (0)		CM (10.00)
BMEII0108	tauB	taurine ABC transporter, ATP-binding protein	6	3 (2)		CM (9.98)
BMEI0635	cbiO	cobalt ABC transporter, ATP-binding protein	6	3 (2)		CM (9.82)
BMEII0704	bfr	bacterioferritin	12	4 (2)	P	C (9.97)
BMEII0178	znuA	zinc ABC transporter, periplasmic zinc-binding protein	9	1 (1)	V	P (9.76)

**Coenzyme transport and metabolism**						

BMEII0589	ribH	riboflavin synthase subunit beta	12	8 (0)		C (9.97)
BMEI2029	ahcY	S-adenosyl-L-homocysteine hydrolase	4	1 (1)		C (9.97)
BMEI1099	cobT	nicotinate-nucleotide--dimethylbenzimidazole phosphoribosyltransferase	2	1 (0)		C (9.97)
BMEI1187	ribH	riboflavin synthase subunit beta	12	8 (0)		C (9.97)

**Cell cycle control, cell division, chromosome partitioning**						

BMEII0470	crcB	crcB family protein	5	1 (0)		CM (10.00)

**General function prediction only**						

BMEII0288	oppF	peptide ABC transporter, ATP-binding protein	6	3 (2)		CM (9.99)
BMEI1081	surE	stationary phase survival protein SurE	8	5 (2)		C (8.96)
BMEI0215	ialA	dinucleoside polyphosphate hydrolase	1	1 (0)		C (9.97)
BMEI0920	mazG	nucleoside triphosphate pyrophosphohydrolase	5	1 (0)		C (8.96)
BMEI1584	ialB	invasion protein B	1	2 (0)	P	U (2.50)
BMEII0355	gal	D-galactose 1-dehydrogenase, putative	5	1 (0)		C (9.97)

**Lipid transport and metabolism**						

BMEI1111	acpXL	acyl carrier protein	5	2 (0)		C (9.97)
BMEI1553	bacA	transport protein	9	5 (4)	V	CM (10.00)
BMEI1475	acpP	acyl carrier protein	5	1 (0)		C (9.26)

**Carbohydrate transport and metabolism**						

BMEII0983	chvE	sugar ABC transporter, periplasmic sugar-binding protein, putative	9	1 (1)		U (5.02)
BMEI1394	manA	mannose-6-phosphate isomerase	5	1 (0)		C (8.96)
BMEI1237	galE	epimerase/dehydratase family protein, putative	6	3 (1)	V	C (8.96)
BMEII0430	eryA	erythritol kinase	5	2 (1)		C (9.26)
BMEI0310	gap	glyceraldehyde-3-phosphate dehydrogenase	5	1 (1)		C (9.97)
BMEII0750	smoK	sugar ABC transporter, ATP-binding protein	6	3 (2)		CM (9.99)
BMEI1416	rfbE	O-antigen export system ATP-binding protein RfbE	6	3 (2)		CM (7.88)
BMEII0940	smoK	sugar ABC transporter, ATP-binding protein	6	3 (2)		CM (9.99)
BMEII0625	ugpB	glycerol-3-phosphate ABC transporter, periplasmic glycerol-3-phosphate-binding protein	1	1 (0)	V	P (9.76)
BMEI0309	pgk	phosphoglycerate kinase	6	1 (1)		C (9.97)
BMEII0899	manB	phosphoglucomutase, putative	10	3 (1)	V	C (9.26)
BMEII0428	eryC	D-erythrulose-1-phosphate dehydrogenase	9	2 (1)	V	C (8.96)
ABM67295	P39	immunogenic 39-kDa protein	15	7 (3)	P	P (9.44)
BMEII0982	rbsA	sugar ABC transporter, ATP-binding protein, putative	5	1 (1)		CM (9.82)
BMEII0355	gal	D-galactose 1-dehydrogenase, putative	5	1 (0)		C (9.97)
BMEI1396	pmm	phosphomannomutase, putative	10	5 (1)	V	C (9.97)
BMEII0145	xylG	D-xylose ABC transporter, ATP-binding protein	6	3 (2)		CM (7.88)
BMEI1886	pgm	phosphoglucomutase	9	2 (1)	V	C (8.96)
BMEII0251	glk	glucokinase	5	1 (1)		C (9.97)

**Intracellular trafficking, secretion, and vesicular transport**						

BMEI1077	yajC	preprotein translocase, YajC subunit	6	1 (1)		CM (9.82)
BMEI1076	secD	protein-export membrane protein, SecD/SecF family	6	1 (1)		CM (10.00)
BMEII0026	virB2	type IV secretion system protein VirB2	9	3 (1)	V	CM (9.46)
BMEII0028	virB4	type IV secretion system protein VirB4	9	4 (1)	V	CM (10.00)
BMEII0029	virB5	type IV secretion system protein VirB5	9	1 (1)	V	U (2.00)
BMEII0032	virB8	type IV secretion system protein VirB8	9	1 (0)	V	U (2.00)
BMEII0025	virB1	type IV secretion system protein VirB1	5	1 (0)	V	E (9.64)
BMEII0034	virB10	type IV secretion system protein VirB10	5	1 (1)	V	U (4.90)

**Unclassified (function unknown)**						

CAA86936	BLS	Brucella lumazine synthase	19	12 (2)	P	C (9.97)
BMEI1305	omp2b	porin Omp2b	16	21 (9)		OM (9.93)
BMEI1306	omp2a	porin Omp2a	13	17 (8)		OM (9.93)
BMEI0135	omp19	lipoprotein Omp19	12	10 (5)	V, P	OM (10.00)
BMEII0017	omp10	lipoprotein Omp10	9	6 (2)	V	OM (10.00)
BMEI0536	omp28	immunoreactive 28 kDa outer membrane protein	19	15 (11)	P	P (10.00)
BMEI0330	opgC	opgC protein, putative	5	1 (1)		CM (10.00)
BMEI0634	crcB	crcB family protein	5	1 (0)		CM (10.00)
BMEI0545	pncA	hypothetical protein	5	1 (0)	V	CM (10.00)

Note: Sub-cellular localization was predicted using PSORTb 3.0 implemented in Vaxign. PSORTb score ranges from 0 (0% probability) to 10 (100% probability). Proteins with a score below 7.44 are classed as unknown. The abbreviations in this table are C (Cytoplasmic), CM (CytoplasmicMembrane), E (Extracellular), OM (OuterMembrane), P (Periplasmic) and U (Unknown).

Based on their roles in host-pathogen interactions, the 140 identified *Brucella *genes associated with *Brucella *vaccines can be classified into three groups: (1) protective antigens, (2) virulence factors, and (3) unknown. Protective antigens elicit a protective immune response, and are frequently used for vaccine development. VO-SciMiner identified all 14 known protective *Brucella *antigens [12]. Virulence factors are expressed and secreted by pathogens and often responsible for causing diseases in the host. Out of the 140 *Brucella *genes associated with *Brucella *vaccines, 46 are known *Brucella *virulence factors based on the updated curation information from the Brucella Bioinformatics Portal (BBP; http://www.phidias.us/bbp) [10,22] (Table 3). In total, 81 *Brucella *genes are neither known protective antigens nor virulence factors. Since these genes have been studied in the context of *Brucella *vaccines, it seems possible that some of them are potential virulence factors and/or protective antigens. For example, *RopB *has not been implicated in inducing immune protection. However, our recent reverse vaccinology analysis using Vaxign [16] found that *Brucella abortus RopB *is a potential *Brucella *adhesin with a probability of 0.815 [12]. This could make *RopB *a promising target for future vaccine development as a potential protective antigen.

A functional analysis of the 140 genes and their subgroups is critical to illustrate their association with *Brucella *vaccine research. *Brucella *genes have been well categorized with the COG system [18]. We performed a functional enrichment analysis identifying significantly over-represented COG categories within all 140 genes or 46 virulence factors. These include: "carbohydrate transport and metabolism", "cell wall, membrane, and envelope biogenesis", "intracellular trafficking, secretion, and vesicular transport", and "posttranslational modification, protein turnover, chaperones" (Table 4).

**Table 4:** COG functional analysis of *Brucella *genes associated with vaccine research

	VO-associated Genes (n = 140)	Virulent Genes (n = 46)
	
COG Description	# of genes (p-value)	# of genes (p-value)
Amino acid transport and metabolism	12 (0.771)	4 (1.000)
Carbohydrate transport and metabolism	19 (0.000*)	6 (0.043*)
Cell cycle control, cell division, chromosome partitioning	1 (1.000)	
Cell motility	1 (1.000)	1 (0.341)
Cell wall/membrane/envelope biogenesis	22 (0.000*)	9 (0.001*)
Coenzyme transport and metabolism	4 (0.820)	
Energy production and conversion	5 (0.843)	1 (0.728)
Inorganic ion transport and metabolism	10 (0.149)	3 (0.475)
Intracellular trafficking, secretion, and vesicular transport	7 (0.003*)	5 (0.000*)
Lipid transport and metabolism	3 (0.627)	1 (1.000)
Nucleotide transport and metabolism	2 (0.769)	1 (1.000)
Posttranslational modification, protein turnover, chaperones	16 (0.000*)	5 (0.025*)
Replication, recombination and repair	14 (0.001*)	3 (0.428)
Secondary metabolites biosynthesis, transport and catabolism	3 (0.750)	1 (0.601)
Signal transduction mechanisms	3 (1.000)	2 (0.357)
Transcription	6 (0.704)	2 (1.000)
Translation, ribosomal structure and biogenesis	5 (0.684)	

Note: * indicates p < 0.05.

Interestingly, the COG category "replication, recombination and repair" is significantly over-represented in the 140 genes (p-value = 0.001); however, this category is not enriched in the virulence factor group (p-value = 0.428). In total, 14 out of 140 *Brucella *genes belong to this category (Table 3). Among them, three genes (*uvrA, mutM*, and *recA*) are known virulence factors. Many of the remaining genes are virulence factors in other pathogens. For example, *XerD*, an enzyme responsible for resolving chromosomal multimers prior to chromosome segregation, is a virulence factor in *E. coli *and *S. aureus *[23,24]. Mutations of *gryA *and *parC *are responsible for generation of quinolone-resistance in many bacteria (e.g., *Pseudomonas aeruginosa*) [25]. These two genes are critical for antibiotics resistance in virulent bacteria. Therefore, it is reasonable to hypothesize that many of the genes in this category may be potential *Brucella *virulence factors.

### Identification of *Brucella *vaccine-associated gene networks

**Figure 4:**
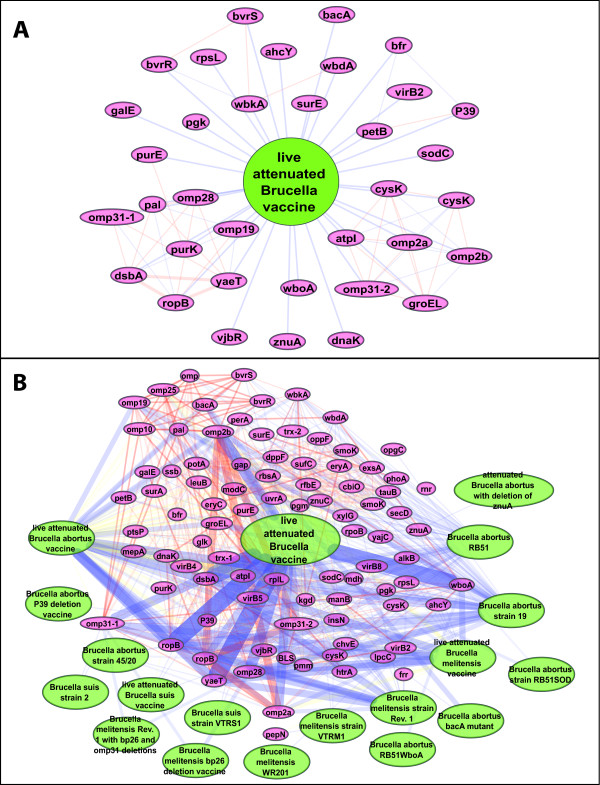
**Networks between genes and live attenuated *Brucella *vaccines identified by VO-SciMiner**. Searches of "live attenuated *Brucella *vaccine" using (A) the PubMed Entrez system and (B) the VO-SciMiner system. Edge color represents the types of association; blue for gene-vaccine association and red for gene-gene association.

Difference in the volume of the retrieved literature can result in a substantial difference in obtainable knowledge. If one is interested in establishing the interaction network of pathogen genes and vaccines, the networks (co-citations of gene and vaccine), based on the PubMed search and the VO-SciMiner search, can be substantially different (Figure 4). Using 74 papers retrieved by PubMed, the interaction network between genes and live attenuated *Brucella *vaccines is extremely limited (Figure 4A). Only 35 genes are associated with live attenuated *Brucella *vaccines. However, when the VO hierarchy of 'live attenuated *Brucella *vaccine' (Figure 2) is considered, 89 (54 more) genes are identified by VO-SciMiner (Figure 4B).

**Figure 5:**
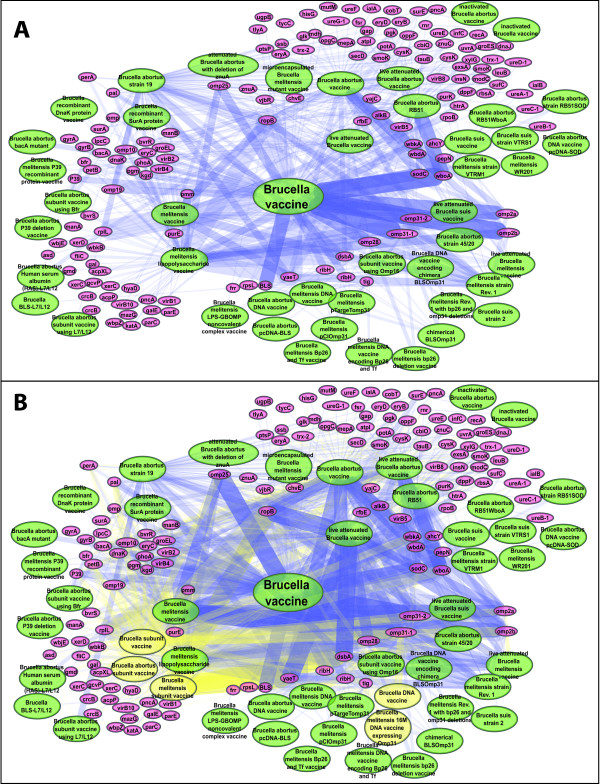
***Brucella *vaccine and gene co-citation network**. All *Brucella *vaccine terms in VO were used. This co-citation network was generated without (A) or with (B) VO hierarchy. VO terms are shown in green and *Brucella *gene in red. The line depth between VO vaccine terms and *Brucella *gene symbols represents the relative number of documents with each pair of VO vaccine terms and *Brucella *genes are co-cited. The terms and edges in yellow were inferred from the VO hierarchy.

Similarly, without VO hierarchy, the *Brucella *vaccine-gene network is relatively sparse (Figure 5A), including only 580 vaccine-gene associations. When VO hierarchy is incorporated, the *Brucella *vaccine-gene network becomes more intense with 1,009 vaccine-gene associations (Figure 5B). This is equivalent to a 74% increase in the number of vaccine-gene associations, resulting in a more comprehensive gene-vaccine association network.

Three types of interactions are observed in the *Brucella *vaccine-gene interaction networks (Figures 4 and 5). The first type of interaction is a vaccine-vaccine association, which results from the asserted *is_a *hierarchy between a vaccine and its parent or child terms as asserted in VO (*e.g*., *Brucella abortus *vaccine and *Brucella abortus *vaccine RB51), or from the inferred VO hierarchy, for example, live attenuated *Brucella *vaccine and *Brucella abortus *vaccine RB51 (Figure 2).

Another type of interaction in the *Brucella *network is vaccine-gene interaction. Many vaccine-gene interactions are based on direct association identified from the literature. Such an example would be an association between *Brucella *RB51 strain vaccine and the *wboA *gene [26]. Alternatively, further vaccine-gene interactions are based on asserted or inferred hierarchy by VO reasoning. Since RB51 is inferred to be a live attenuated *Brucella *vaccine, an inferred association between live attenuated *Brucella *vaccine and the *wboA gene *can be made. Indeed, based on the finding of a *wboA *mutation in RB51, many live attenuated *Brucella *vaccine candidates, such as VTRS1 and VTRM1 [27], have been developed by mutating the *wboA *sequence in various wild type *Brucella *strains.

The present study also reveals that four *B. suis *vaccine-gene interactions are isolated from the other interactions, suggesting that these vaccine-gene interactions were only studied in the context of *B. suis*, but not for other *Brucella *species. The inferred vaccine-gene associations are potentially valuable for systematic analysis of genes associated with less-specific type of vaccines (*e.g*., live attenuated *Brucella *vaccines) instead of specific vaccines such as RB51.

The last type of interaction in the *Brucella *network is gene-gene interaction. The obvious gene-gene interactions in a network are found between those genes co-cited in the same paper(s). In addition, many new gene-gene interactions can be inferred through their association with the same vaccines. In a gene-gene interaction network under the scope of live attenuated *Brucella *vaccine based on PubMed search (Figure 4A), two genes in a two-component system, *bvrR *and *bvrS *are associated, and are linked to one gene, *wbkA *(Figure 4A). When the vaccine hierarchy was used in VO-SciMiner, eight more genes were detected that are associated with *bvrR *and *bvrS *under the same scope of live attenuated *Brucella *vaccine. VO-SciMiner also detects what specific live attenuated *Brucella *vaccines (*e.g*., *B. abortus *vaccine RB51) interact with a gene (*e.g*., *wboA*). New hypotheses can also be generated based on the gene-gene network under a *Brucella *vaccine domain. For example, *Brucella bvrS *and *bvrR *are only associated directly with *B. abortus *vaccines (Figure 4A and 4B). However, many genes (*e.g*., *wbdA *and *wbkA*) that interact with *bvrS *and *bvrR *are also associated with live attenuated *B. melitensis *and *B. suis *vaccines. Therefore, it is reasonable to hypothesize that the mutants of *bvrR *or *bvrS *gene in *B. melitensis *and *B. suis *may also be candidates for live attenuated *Brucella *vaccines.

### A web server for browsing and analyzing VO-SciMiner literature mining results

**Figure 6:**
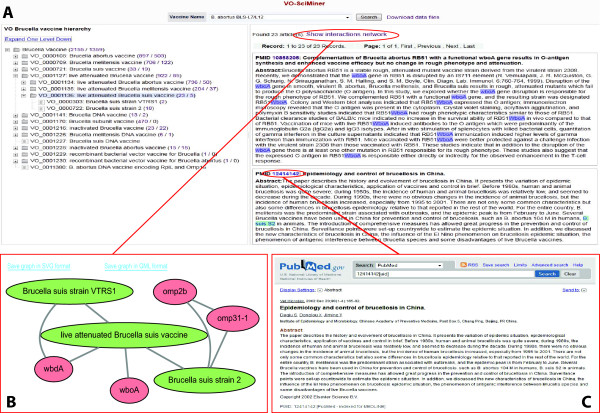
**Screen shot of VO-SciMiner webpage**. Two panels are illustrated in this web page with "VO_0001136: live attenuated Brucella suis vaccine" selected. The left panel shows the hierarchical structure of the ontology term '*Brucella *vaccine' and its child terms in VO. Two numbers are displayed next to each of the VO terms. The first number represents the total number of search hits including the research results from all the child terms (if any) and the second number is the number of hits for the current term. Once either number is clicked, the abstract details are displayed on the right panel. The identified VO terms and *Brucella *genes are highlighted.

A web-based application of VO-SciMiner-based *Brucella *paper indexing has been developed (http://www.violinet.org/vo-sciminer). This webpage provides two ways to explore the *Brucella *vaccine literature (Figure 6A). The first option is to select a vaccine using the dropdown list. A list of publications related to the selected vaccine will be displayed on the lower right panel when the search button is clicked. For each publication, VO-SciMiner lists the PubMed ID, the title, and an abstract if available. In the abstract section, all the related vaccines and genes are highlighted and linked to a page containing detailed information of corresponding vaccines and genes. Another way to select a vaccine is by navigating through the hierarchical structure of different vaccines on the lower left panel. This panel lists all the *Brucella *vaccines on an inferred tree structure. If available, two numbers will be shown for each node. The first number is the total number of publications related to this node and any of its child nodes. The second number shows the number of publications related only to this node. If any of the numbers are clicked, the lower right panel will display a detailed list of publications. A hyperlink 'Show interactions network' will appear at the top of the screen. This will bring up a new display of the vaccine and gene interaction network (Figure 6B).

## Discussion

The present study demonstrates that the application of Vaccine Ontology in literature mining enhances the retrieval of *Brucella *vaccine literature. Term variation rules, inclusion vaccine synonyms, and the inference of vaccine terms through the hierarchy increases the amount of literature that can be retrieved. VO-based SciMiner is capable of identifying up to 15 times more *Brucella *vaccine papers than the typical PubMed search using the Entrez system. The increase in the retrieved literature results in the generation of a comprehensive co-citation network of *Brucella *genes and *Brucella *vaccines, revealing key vaccine-gene associations and provides potential valuable opportunities for the development of new vaccines.

To our knowledge, this study is the first manuscript that compares MeSH and specific domain ontology (*e.g*., Vaccine Ontology in this case) for literature indexing. The results show that VO can be used to improve literature searching for *Brucella *vaccines. While MeSH provides reasonable searching specificity, its level of sensitivity may be very low for many biomedical research areas (*e.g*., vaccines). For example, "*Brucella *vaccine" is currently one of the lowest level (the most specific) terms in MeSH, and no individual *Brucella *vaccines such as RB51 are indexed by MeSH. In contrast, VO-based SciMiner contains detailed vaccine terminologies in a controlled hierarchical structure, allowing searches across the hierarchy, thereby achieving a very significant recall.

Therefore, it is possible to substantially increase PubMed searching sensitivity by incorporating ontology information. Increased sensitivity in turn will result in increasing the amounts of information obtained on any given search topic. As illustrated in the gene-vaccine networks in Figure 4, the level of obtainable information from papers either retrieved by PubMed (A) and VO-SciMiner (B) is substantial. Based on the hierarchy structure of VO, the VO-based approach can retrieve a more comprehensive network. Therefore, it is possible to use a community-based ontology (*e.g*., VO) to replace, or enhance, a part of a MeSH-based document retrieval system such as PubMed in some domains (*e.g*., in vaccine).

Using ontologies to support literature mining has become an active area of research over the last decade. Multiple text-mining tools and web-resources, including GoPubMed [28], SEGOPubMed [29], and GoWeb [30] have been developed to link PubMed papers to ontologies, particularly Gene Ontology, within the biomedical data. Using the domain knowledge and hierarchical organization, these tools allow users to perform ontology-based PubMed document browsing. However, they are limited by lack of coverage in the vaccine domain as in the PubMed Entrez search system. BioPortal (http://bioportal.bioontology.org), a steadily growing online resource developed by the National Center for Biomedical Ontology (NCBO), currently indexes multiple biomedical public resources. These include gene expression and protein-protein interaction data sets, which are comprised of over 250 ontologies and 4 million ontology terms. The BioPortal provides a convenient web interface. Unfortunately, PubMed biomedical literature is not supported in the BioPortal yet. In this study, we demonstrated that the newly developed VO provides a powerful system for ontology-based vaccine literature indexing, through the dictionary- and rule-based VO-SciMiner system. It offers the further advance of a significantly enhanced recall and precision. The dictionary- and rule-based literature mining system for optimization of a vaccine term searching is not presently found in the BioPortal or any other literature mining system.

In an effort to improve vaccine literature mining and to support the vaccine research community, VO-SciMiner has been integrated into the VIOLIN vaccine research database and data analysis system (http://www.violinet.org)[11]. VIOLIN includes all the vaccine literature from PubMed and provides multiple tools for navigating various vaccines and relevant literature. These tools include (1) Litesearch providing basic vaccine literature keyword searching feature; (2) Vaxmesh, a MeSH-based visualization tool for displaying the MeSH hierarchy and the numbers of vaccine papers associated with each MeSH terms; (3) Vaxpresso, a VIOLIN vaccine literature mining program powered by Textpresso natural language processing (NLP) program [31], for identifying genes and proteins in literature data. Initially, we intended to integrate VO into our Vaxpresso. However, Textpresso, the base system of Vaxpresso, had a limitation of slow-execution since it is a plain text file-based system rather than a relational database-based program. Compared to these existing VIOLIN text mining tools, VO-SciMiner is a significant extension because it is the first NLP literature mining program that integrates VO terms and VO hierarchy for efficient vaccine literature indexing. This study also represents the first effort to apply VO to studying the interaction network between vaccines and microbial genes.

In addition to improving the retrieval of *Brucella *vaccine-related papers by incorporating VO into SciMiner, SciMiner was extended to identify *Brucella *pathogen genes reported in the literature. Comprehensive co-citation networks of *Brucella *vaccines and *Brucella *genes were generated as illustrated in Figures 4 and 5. These comprehensive networks not only illustrate direct gene-vaccine associations co-cited in the literature, but also reveal indirect associations inferred through the VO hierarchy. These literature-based networks can enhance vaccine development, since they allow the generation of additional hypotheses. As demonstrated in this paper, many *Brucella *genes are potential new protective antigens and/or virulence factors. Protective antigens are defined as those genes that are able to induce protection *in vivo*. Mutation of a virulence factor gene in *Brucella *results in an attenuated mutant strain, which can be a potential live attenuated vaccine. Therefore, it is suggestive that genes identified by the presently described methodology can serve possible targets for development of new *Brucella *vaccines.

It should be noted that the text mining approach implemented in VO-SciMiner has its own limitations. The co-citation-based VO-SciMiner is still in an early stage of being applied to the field of vaccinology research. The identification of potential new gene-vaccine associations based on a text mining method may include false-positives due to excellent but still imperfect performance (an F-measure of 95%). Besides, co-citation of both a gene and a vaccine in the same document does not necessarily mean that they are functionally associated. Therefore, any interesting association will require careful consideration and experimental investigation. In spite of these limitations, text mining studies have proven useful in identifying previously unknown knowledge such as predicted combinatorial binding of transcriptional factors to regulatory elements [32], and reconstructing protein-protein interactions and pathways [33].

Our current study has also generated predictions that form new hypotheses for further experimental studies. To make our current approach more robust, the gene-vaccine text mining method may be improved in the future by retrieving additional data, including the types of interactions, the animal or cell types used, and specific experimental designs and conditions. Furthermore, the text mining prediction approach becomes more powerful and specific when it is integrated with other computational and experimental methods such as microarray and proteomics studies [34-36].

*Brucella *vaccines were used as examples in this study. In the near future, other bacterial vaccines will be added to the VO-SciMiner system. Incorporating additional rules for species other than *Brucella *may be challenging if other bacterial vaccines have substantially different structures than *Brucella *VOs. However, the *Brucella *case study as presented provides a general strategy and framework to address these issues. The VO-SciMiner indexing strategy can also be used to improve other gene interaction network studies. For example, previous studies presented the retrieval of an IFN-γ and vaccine-mediated immune network based on a genome-wide centrality-based literature discovery method [37], which was improved by application of VO [38]. However, it is suggestive that further improvements may be made in the identification of vaccine and gene entities contained in the literature using the VO-SciMiner indexing approaches. The indexing of human and mouse genes for studying the interactions between *Brucella *vaccines and host genes is currently under investigation. The approach taken in this study is generic and can be applied to analyze other vaccines listed in VO.

## Conclusions

We have developed a methodology that incorporates Vaccine Ontology into literature mining to improve relevant paper retrieval in the domain of vaccine research. To test our methodology, *Brucella *vaccines were used. Our analyses indicate that the VO-based SciMiner method substantially increases retrieval of associated data, leading to an improved analysis and understanding of the vaccine-gene networks.

## List of abbreviations used

MeSH: Medical Subject Headings; VO: Vaccine Ontology; OBO: Open Biological and Biomedical Ontologies; BFO: Basic Formal Ontology; COG: Clusters of Orthologous Groups.

## Authors' contributions

JH conceived and coordinated the project, developed the VO-SciMiner, generated all figures and tables, and drafted the manuscript. ZX implemented VO-SciMiner web server, and participated in writing of the manuscript. ELF supervised the project and edited the manuscript. YH conceived and supervised the project, served as the domain expert in interpretation of the results, and drafted and edited the manuscript. All authors read and approved the final manuscript.
